# A Novel Risk Model Based on Autophagy-Related LncRNAs Predicts Prognosis and Indicates Immune Infiltration Landscape of Patients With Cutaneous Melanoma

**DOI:** 10.3389/fgene.2022.885391

**Published:** 2022-04-29

**Authors:** Qi Shu, Yi Zhou, Zhengjie Zhu, Xi Chen, Qilu Fang, Like Zhong, Zhuo Chen, Luo Fang

**Affiliations:** ^1^ The Cancer Hospital of the University of Chinese Academy of Sciences (Zhejiang Cancer Hospital), Institute of Basic Medicine and Cancer (IBMC), Chinese Academy of Sciences, Hangzhou, China; ^2^ Department of Pharmacy, First People’s Hospital of Linping District, Hangzhou, China

**Keywords:** cutaneous melanoma, autophagy-related lncRNAs, immune infiltration landscape, risk model, prognosis

## Abstract

Cutaneous melanoma (CM) is a malignant tumor with a high incidence rate and poor prognosis. Autophagy plays an essential role in the development of CM; however, the role of autophagy-related long noncoding RNAs (lncRNAs) in this process remains unknown. Human autophagy-related genes were extracted from the Human Autophagy Gene Database and screened for autophagy-related lncRNAs using Pearson correlation. Multivariate Cox regression analysis was implemented to identify ten autophagy-related lncRNAs associated with prognosis, and a risk model was constructed. The Kaplan–Meier survival curve showed that the survival probability of the high-risk group was lower than that of the low-risk group. A novel predictive model was constructed to investigate the independent prognostic value of the risk model. The nomogram results showed that the risk score was an independent prognostic signature that distinguished it from other clinical characteristics. The immune infiltration landscape of the low-risk and high-risk groups was further investigated. The low-risk groups displayed higher immune, stromal, and ESTIMATE scores and lower tumor purity. The CIBERSORT and single sample gene set enrichment analysis (ssGSEA) algorithms indicated a notable gap in immune cells between the low- and high-risk groups. Ten autophagy-related lncRNAs were significantly correlated with immune cells. Finally, Gene Set Enrichment Analysis (GSEA) and Gene Set Variation Analysis (GSVA) results demonstrated that autophagy-related lncRNA-mediated and immune-related signaling pathways are crucial factors in regulating CM. Altogether, these data suggest that constructing a risk model based on ten autophagy-related lncRNAs can accurately predict prognosis and indicate the tumor microenvironment of patients with CM. Thus, our study provides a new perspective for the future clinical treatment of CM.

## Introduction

Cutaneous melanoma (CM) is the most common subtype of melanoma, accounting for 5% of all skin cancers and 75% of deaths globally (Rebecca et al., 2020). There were 320,000 newly diagnosed cases and 57,000 deaths from cutaneous melanoma worldwide in 2020, indicating that cutaneous melanoma has become a major challenge to human health (Bray et al., 2018). Notably, the 5-year survival rate of patients with metastatic melanoma is less than 23% and is often accompanied by brain metastases, which seriously affects the quality of life of patients (Arheden et al., 2019). Melanoma is primarily a malignant transformation of melanocytes derived from neural crest stem cells (NCSCs) that produce melanin in the skin (Shakhova, 2014). Metastatic melanoma was regarded as an incurable disease until targeted immunotherapy strategies were approved in 2011 and 2014 (Dummer et al., 2018). Therefore, exploring new disease markers and therapeutic targets is vital for the treatment of CM.

Autophagy is an important physiological function of organisms and acts as the main cellular clearance mechanism by transporting damaged organelles and misfolded or mutated proteins to lysosomes for degradation (Glick et al., 2010; Mizushima and Komatsu, 2011; Antunes et al., 2018). However, imbalanced autophagy regulation is associated with various human diseases, such as neurodegenerative, inflammation, cardiovascular, and metabolic diseases (White et al., 2015; Ravanan et al., 2017; Guo et al., 2018; Racanelli et al., 2018; Wang et al., 2019). In addition, Li et al. (2017) reported that autophagy plays a dual role in tumor resistance and that regulating autophagy can improve the sensitivity of tumor cells to chemotherapeutics. Nazio et al. (2019) indicated that autophagy could affect the generation, differentiation, plasticity, migration/invasion, and immune resistance of cancer stem cells, and thus is a possible pivotal treatment target for tumor therapy. Yamazaki et al. found that autophagy could also influence the tumor microenvironment to promote tumor proliferation and differentiation (Yamazaki et al., 2021). Autophagy has broad application prospects for the treatment of tumors. A previous study suggested that autophagy is involved in the progression and chemoresistance of melanoma (Rather et al., 2020). Lazova et al. (2010) showed that melanoma cells display high levels of autophagy and may exist as a constitutive state of invasive and metastatic melanoma cells. Therefore, discovering autophagy-related biomarkers is critical for the early diagnosis and prognosis of patients with CM.

Long noncoding RNAs (lncRNAs) are defined as transcripts greater than 200 nucleotides in length without an evident protein-coding function (Ransohoff et al., 2018). LncRNAs have been reported to play essential roles in many aspects of gene expression during development and disease (Sanchez Calle et al., 2018). LncRNAs are abnormally expressed in various diseases, including metabolic and cardiovascular diseases, as well as in tumors (Li et al., 2018; Chi et al., 2019). In addition, several lncRNAs have been demonstrated to be potential biomarkers and therapeutic targets for tumor diagnosis. For example, Nissan et al. (2012) discovered that CCAT1 is significantly overexpressed in colon cancer tissues while another study suggested that MALAT1 may be a prognostic biomarker in breast and lung cancers (Sun and Ma, 2019). Furthermore, Ying et al. (2013) demonstrated that the downregulation of MEG3 could activate autophagy in bladder cancer cells and promote their proliferation. However, the role of autophagy-related lncRNAs in CM remains largely unknown.

The tumor microenvironment (TME) incorporates blood vessels, extracellular matrix, fibroblasts, immune cells, and signaling molecules, widely implicated in tumorigenesis progression (Spill et al., 2016; Arneth, 2019). LncRNAs are expressed in a highly lineage-specific manner and control the differentiation and function of innate and adaptive cell types, which affects the immune microenvironment (Chen et al., 2017). In a study of human lymphocyte populations, more than 70% of the expressed lncRNAs were specific to a single lymphocyte subset, indicating that the regulation of genes by lncRNAs might be critical for spatial aspects of the immune response (Ranzani et al., 2015). Moreover, lncRNAs regulate the development of inflammation in response to activation of the immune system (Castellanos-Rubio et al., 2016). Recently, increasing evidence has indicated that autophagy-related lncRNAs greatly influence the immune microenvironment in multiple tumors and can indicate the immune status of different populations (Chen et al., 2021). However, the association between autophagy-related lncRNAs and the immune microenvironment in CM remains unclear.

The construction of prognostic models using bioinformatics and genome sequencing technologies to identify potential prognostic biomarkers has recently attracted increasing attention (Liu and Liu, 2020). In this study, after analysis of The Cancer Genome Atlas (TCGA) database, we systematically investigated the association between autophagy-related lncRNAs and clinicopathological characteristics of patients with CM. A novel risk model was constructed based on ten autophagy-related lncRNAs, and the ability of autophagy-related lncRNAs to predict the prognosis of patients with CM was assessed. In addition, the immune microenvironment of patients with CM was comprehensively analyzed, and the potential signaling pathways involved were explored. In summary, the results of this study provide a fresh perspective and insights regarding potential strategies for treatment of patients with CM.

## Materials and Methods

### Data Collection

Clinical data and gene expression matrices for patients were downloaded from The Cancer Genome Atlas database (TCGA) (https://portal.gdc.cancer.gov/) (Wang et al., 2016). The normalized transcriptome gene expression matrix (RNA-Sep, FPKM format) of 458 patients with CM were extracted and analyzed. Perl scripts were used to extract the gene expression matrix for further analyses. LncRNAs and protein-coding genes were annotated and classified using the Ensembles human genome browser GRCh38.p13 (http://asia.ensembl.org/index.html) (Cunningham et al., 2019). Clinical data included survival time, survival status, age, sex, grade, T stage, and N stage. All data and clinical information involved in this study were obtained from a public database. Approval from the ethics committee and written informed consent from patients were not required.

### Identification of Autophagy-Related LncRNAs and Construction of the LncRNAs-mRNAs Co-Expression Network

A total of 232 human autophagy-related genes were extracted from the Human Autophagy Gene Database (http://www.autophagy.lu/) (Chen et al., 2020). In addition, Pearson’s correlation analysis was used to determine the correlation between lncRNAs and autophagy-related genes. The threshold was set as the absolute value of the correlation coefficient >0.3, *p*-value < 0.001 (|R^2^| > 0.3, *p* < 0.001). The autophagy-related lncRNA and autophagy-related gene co-expression network were constructed using the Cytoscape software (version 3.7.2, http://www.cytoscape.org/).

### Construction of Prognostic Model and Calculation of Risk Score

Univariate Cox regression analysis was used to identify candidate lncRNAs that were significantly associated with the survival rate of patients with CM. The hazard ratio (HR) was used to determine whether lncRNAs were risk factors (HR > 1) or protective factors (HR < 1). Subsequently, the candidate autophagy-related lncRNAs were subjected to multivariate Cox regression analysis to evaluate whether their contribution was an independent prognostic factor for patient survival probability. The risk scores of each patient were calculated according to the prognostic signature using the following formula: $\mathrm{risk}\;\mathrm{score}=\sum_{i=1}^{n}Coef\left(i\right)\;\;xx\left(i\right)$ , where *Coef(i)* represents the correlation regression coefficient of autophagy-related lncRNAs, and *x(i)* represents the expression level of autophagy-related lncRNAs.

### Evaluation of Prognostic Characteristics and Validation of Risk Model

For all cohorts, patients with CM were divided into high-risk and low-risk groups according to the median risk score. The Kaplan–Meier survival curve was utilized to show the survival probability of patients with CM in high-risk and low-risk groups *via* “survival” R packages. Principal component analysis (PCA) was applied to show the distribution pattern of the autophagy-related lncRNAs between the high-risk and low-risk groups. Subsequently, a scatter dot plot was constructed to determine the relationship between survival time and risk scores. The nomograms of five clinicopathological characteristics and risk scores were constructed using the “rms” R packages to predict the 3- and 5-year survival probability of patients with CM. All variables were calculated using Cox regression analysis, with lower scores indicating a better prognosis. Calibration plots and consistency (C-index) are commonly used to assess nomogram accuracy. As a model parameter, the C-index was positively correlated with nomogram accuracy. The calibration plot was another parameter used to verify the accuracy of the nomogram: a straight line closer to 45° indicated better predictive power. Additionally, to validate the accuracy of the risk model constructed using risk scores, patients were randomly divided into training (229 samples) and validation cohorts (229 samples). Patients in the training and validation cohorts were stratified into low-risk and high-risk groups, respectively, according to the median risk score value.

### Gene Set Enrichment Analysis and Gene Set Variation Analysis

The Gene Set Enrichment Analysis (GSEA; http://www.broadinstitute.org/gsea) was employed to identify differentially expressed gene sets’ signaling pathways between high-risk and low-risk groups. One thousand permutations were used and screened using the largest and smallest gene set filters of 500 and 15 genes, respectively. FDR <0.05 and p.adj <0.05 were considered to show significant difference. Moreover, the activity of each KEGG term in each patient was evaluated *via* gene set variation analysis (GSVA) using the “GSVA” R packages.

### Immune Infiltration Landscape Analysis

To estimate the immune infiltration landscape of the high-risk and low-risk groups, CIBERSORT and ssGSEA algorithms were used to determine the proportion of each immune cell. The gene expression of immune cells is based on “CIBERSORT R script v1.03,” with the algorithm run using the 22 signatures for 1,000 permutations. A single sample gene set enrichment analysis (ssGSEA) algorithm was performed to assess the infiltration level of 23 types of immune cells *via* the “GSVA” R packages. In addition, the immune, stromal, and ESTIMATE scores and tumor purity of patients with CM were calculated *via* “ESTIMATE” R packages (Yoshihara et al., 2013). Finally, correlation analysis was performed to determine the association between immune cells and autophagy-related lncRNAs *via* “ggplot2” R packages.

### Statistical Analysis

All statistical analyses were performed using R software (version 4.1.0). Correlation analyses between the two variables were performed using the Pearson test, with *p* < 0.05 considered significantly different. Differential functions were analyzed using the Wilcoxon rank-sum test between the two groups, and statistical significance was set at *p* < 0.05.

## Results

### Prognostic Autophagy-Related lncRNAs

**FIGURE 1:**
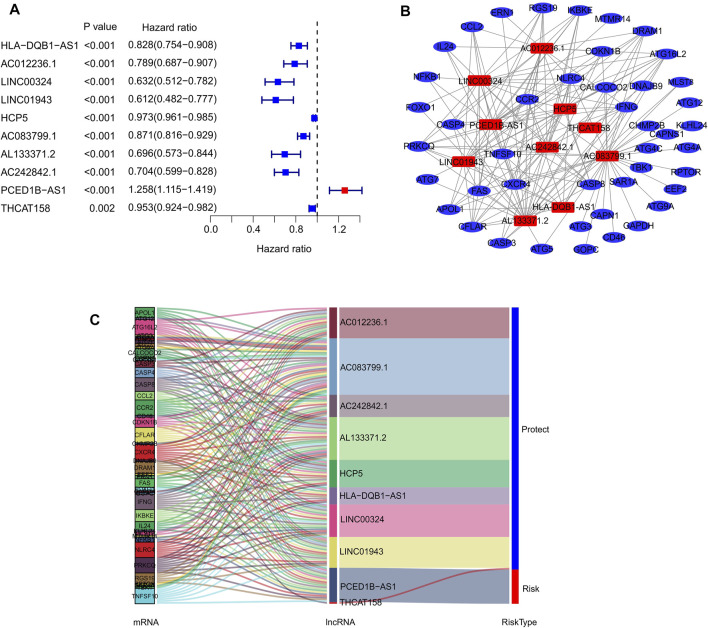
Identification of prognostic autophagy-related lncRNAs. **(A)** Multivariate Cox analysis of prognostic autophagy-related lncRNAs. **(B)** Network analysis of autophagy related lncRNAs and autophagy mRNAs. **(C)** The Sankey diagram shows the connection degree between autophagy mRNAs and autophagy-related lncRNAs.

In the present study, 14,142 lncRNAs were identified based on the RNA-Seq matrix of CM. To screen lncRNAs related to autophagy, 232 autophagy-related genes were extracted to construct the autophagy-related gene lncRNA co-expression network from the Human Autophagy Gene Database. Ultimately, 813 autophagy-related lncRNAs were identified in this study. The multivariate Cox regression analysis results demonstrated that ten autophagy-related lncRNAs had independent prognostic values (Figure 1A). Next, co-expression network analysis was carried out to investigate autophagy-related genes that interacted with the ten autophagy-related lncRNAs (Figure 1B; Supplementary Table S1). The Sankey diagram showed the interactions between autophagy mRNAs and autophagy-related lncRNAs (Figure 1C). AC012236.1, LINC00324, HCP5, THCAT158, LINC01943, AC242842.1, AC083799.1, HLA-DQB1-AS1, and AL133371.2 were protective factors, whereas PCED1B-AS1 was a risk factor for patients with CM.

### Risk Model Construction of Autophagy-Related LncRNAs

**FIGURE 2:**
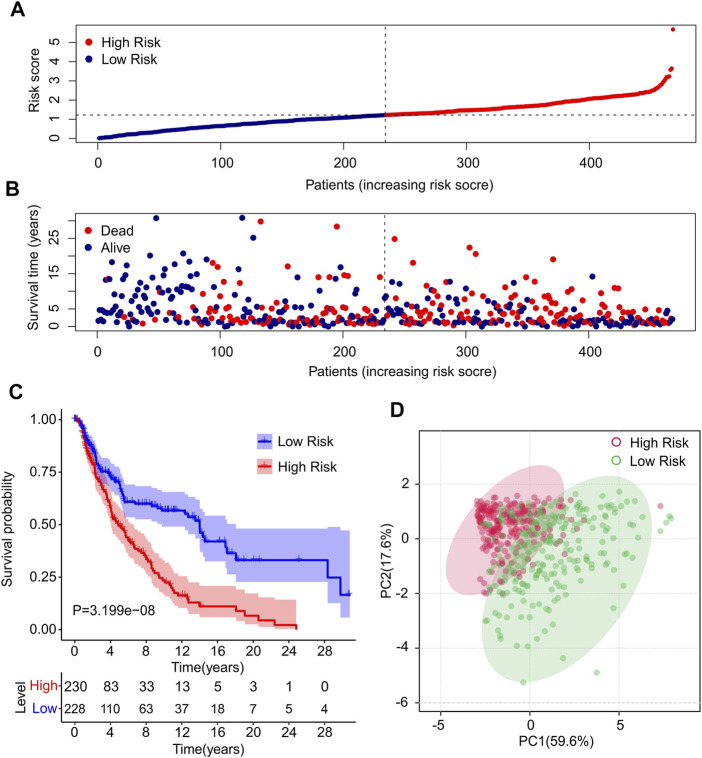
Construction of prognostic characteristics and risk model in CM patients. **(A)** Distribution of risk scores in high- and low-risk CM patients based on the autophagy-related lncRNAs prognostic signature. **(B)** The scatter dot plot illustrates the correlation of survival time and risk scores of CM patients based on the autophagy-related lncRNAs prognostic signature. **(C)** The Kaplan-Meier survival curve analysis shows that the survival time of CM patients with high-risk scores is significantly shorter than those with low-risk scores. **(D)** Principal components analysis (PCA) shows a significant separation between high-risk and low-risk groups based on 10 autophagy-related lncRNAs.

Risk scores for each patient were constructed based on the expression of ten autophagy-related lncRNAs and regression coefficients. The risk scores were calculated using the following formula: risk scores = (−0.186 × expression level of AC012236.1) + (−0.22 × expression level of LINC00324) + (−0.01 × expression level of HCP5) + (−0.031 × expression level of THCAT158) + (−0.031 × expression level of LINC01943) + (−0.231 × expression level of AC242842.1) + (−0.07 × expression level of AC083799.1) + (−0.087 × expression level of HLA-DQB1-AS1) + (−0.22 × expression level of AL133371.2) + (0.229 × expression level of PCED1B-AS1). The patients were divided into low- and high-risk groups based on the median risk scores. The risk score distribution between both groups was calculated based on the autophagy-related lncRNA prognostic signature, and the scatter dot plot showed that the patients’ survival time was inversely correlated with the risk scores (Figures 2A,B). Kaplan–Meier survival curve analysis indicated that the survival time of patients with high-risk scores was significantly lower than that of patients with low-risk scores (*p* = 3.199e-08, Figure 2C). Principal component analysis (PCA) based on ten prognostic autophagy-related lncRNAs showed a clear separation between the high- and low-risk groups (Figure 2D).

In addition, the expression of ten autophagy-related lncRNAs showed a significant difference between the low- and high-risk groups (Supplementary Figure S1). The expression of AC012236.1, LINC00324, HCP5, THCAT158, LINC01943, AC242842.1, AC083799.1, HLA-DQB1-AS1, and AL133371.2 in high-risk groups was significantly lower than that in the low-risk groups. Notably, the expression of PCED1B-AS1 was higher in the high-risk groups than in the low-risk groups.

### Risk Model in Training Cohort and Validation Cohort

**FIGURE 3:**
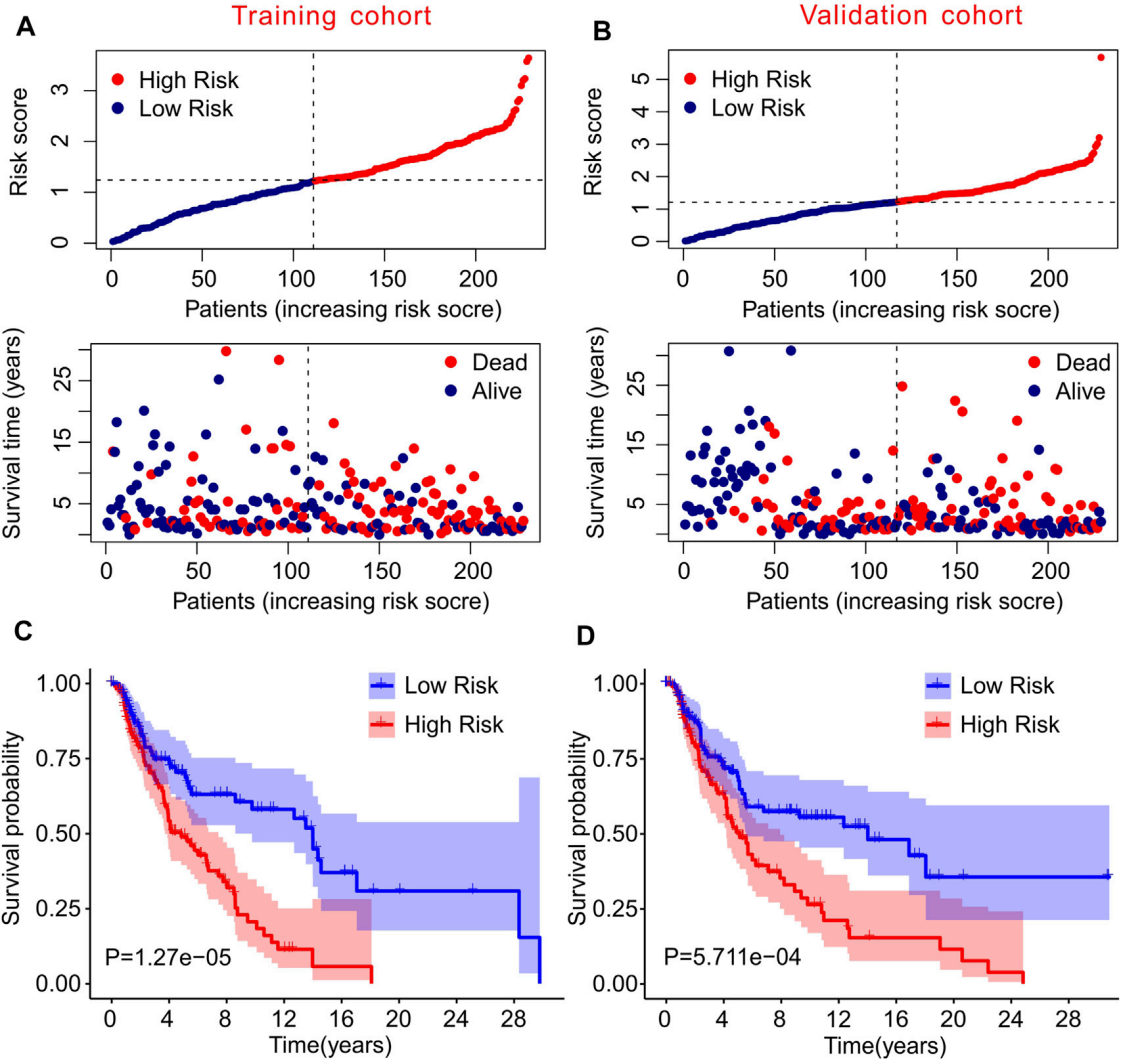
Risk model of training cohort and validation cohort. **(A,B)** The distribution of risk scores and scatter dot plot of CM patients based on the autophagy-related lncRNAs prognostic signature in training cohort and validation cohort. **(C,D)** The survival curve analysis shows that the survival time in CM patients with high-risk scores is significantly shorter than those with low-risk scores in training cohort and validation cohort.

To further confirm the practicability and reliability of the risk model based on autophagy-related lncRNAs, patients with CM were divided into training and validation cohorts. According to the median value of the risk score, patients in both cohorts were stratified into low- and high-risk groups. As shown in Figure 3A, patients with CM were ranked according to the risk scores calculated based on the prognostic signature of autophagy-related lncRNAs, and the scatter dot plot indicated that the patients’ survival time was inversely correlated with the risk scores. Patients in the validation cohort were also ranked according to their risk scores, and the scatter dot plot showed that high-risk groups were characterized by more deaths and shorter survival times (Figure 3B). In addition, Kaplan–Meier survival curve analysis suggested that the survival probability of the low-risk groups was significantly higher than that of the high-risk group in both cohorts (*p* = 1.27e-05, Figures 3C,D). These results demonstrated that constructing a risk model based on autophagy-related lncRNAs is stable and reliable.

### Survival Curve Analysis Based on Autophagy-Related LncRNAs

**FIGURE 4:**
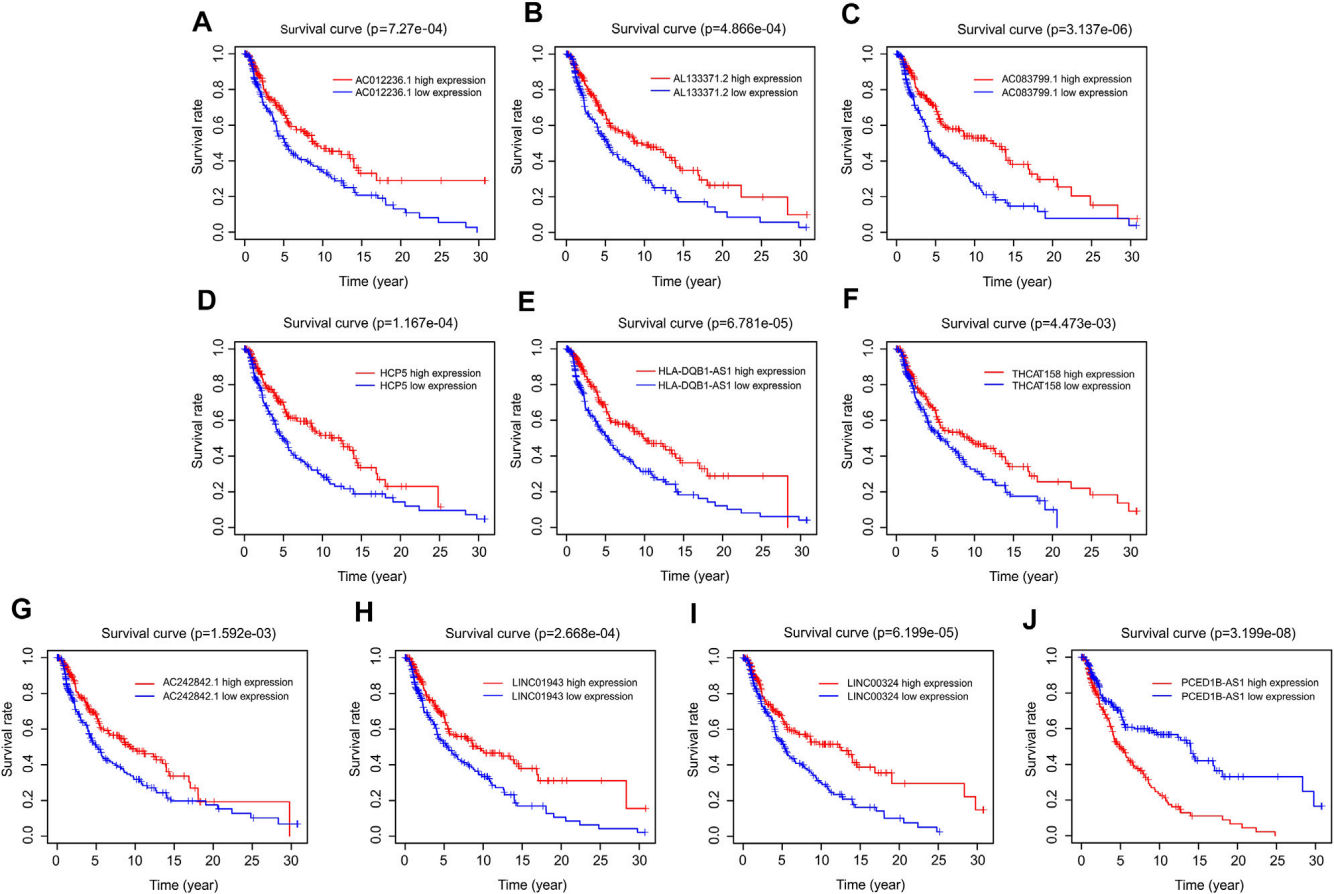
Survival curve analysis of autophagy-related lncRNAs. The Kaplan-Meier survival curve analysis of **(A)** AC012236.1, **(B)** AL133371.2, **(C)** AC083799.1, **(D)** HCP5, **(E)** HLA-DQB1-AS1, **(F)** THCAT158, **(G)** AC242842.1, **(H)** LINC01943, **(I)** LINC00324, **(J)** PCED1B-AS1.

The prognostic survival curves of the ten autophagy-related lncRNAs were evaluated in a subsequent study. As shown in Figure 3, the survival curve showed a notable difference in the survival rate between low and high expression of the ten autophagy-related lncRNAs. The survival rates of the high expression groups were significantly higher than that of the low expression groups, including AC012236.1 (*p* = 7.27e-04), AL133371.2 (*p* = 4.866e-04), AC083799.1 (*p* = 3.137e-06), HCP5 (*p* = 1.167e-04), HLA-DQB1-AS1 (*p* = 6.781e-05), THCAT158 (*p* = 4.473e-03), AC242842.1 (*p* = 1.592e-03), LINC01943 (*p* = 2.668e-04), LINC00324 (*p* = 6.199e-05) (Figures 4A–I). The survival rate of CM patients with high expression PCED1B-AS1 was significantly lower than that of patients with low expression (Figure 4J). Overall, these results suggest that the expression of the ten autophagy-related lncRNAs is notably associated with the survival prognosis of patients with CM.

### Correlation Analysis of Autophagy-Related LncRNAs Risk Scores With Clinicopathological Characteristics

**FIGURE 5:**
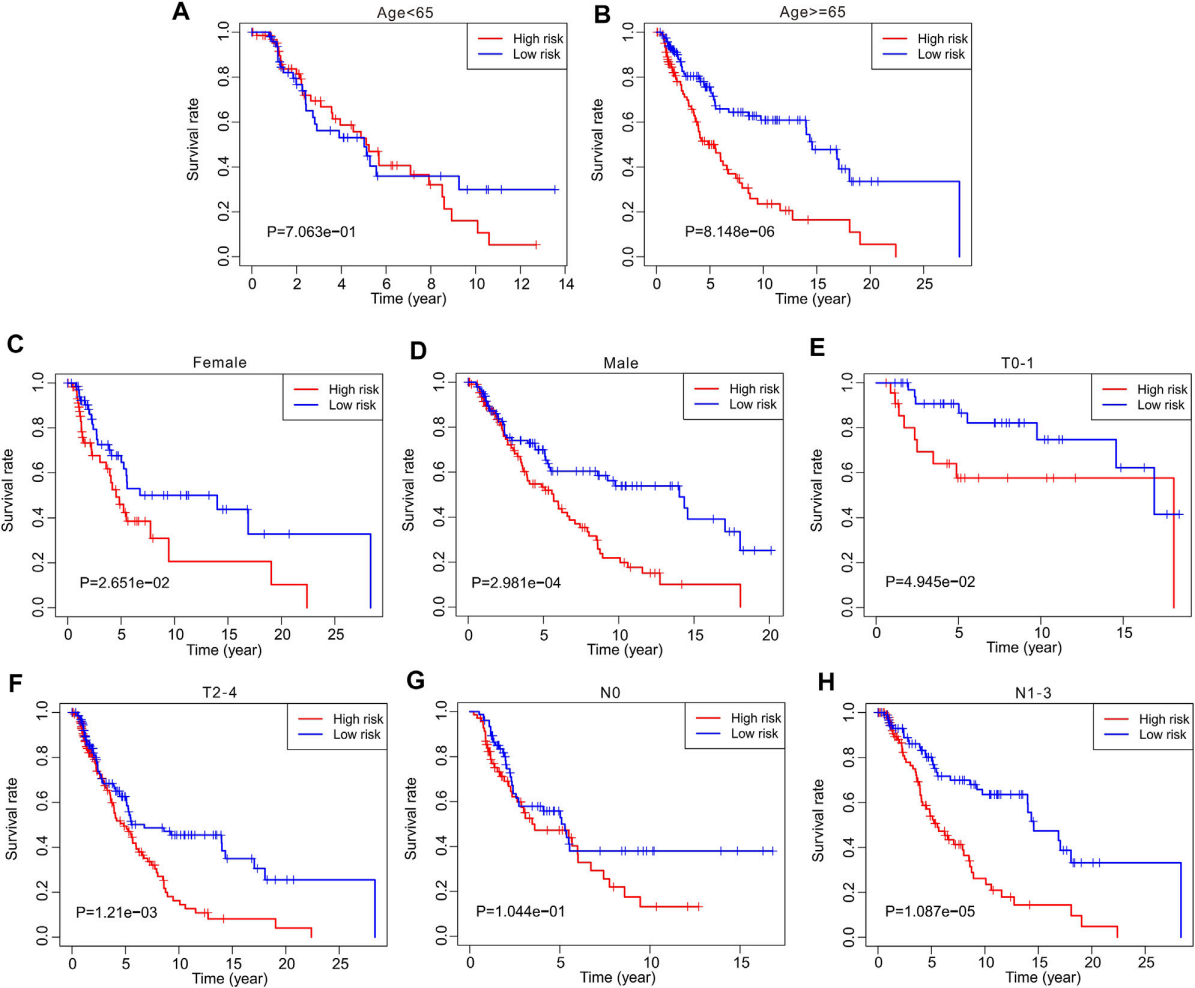
The Kaplan-Meier survival curve analysis of clinicopathological characteristics. The Kaplan-Meier survival curve analysis shows the overall survival rate of low- and high-risk in CM patients stratified by **(A,B)** Age <65 vs. ≥65, **(C,D)** Female vs. Male, **(E,F)** T 0–1 vs. T 2–4, **(G,H)** N 0 vs. N 1–3.

Thereafter, stratified analysis was performed to investigate the prognostic value of autophagy-related lncRNAs. In this study, the patients were classified according to age (<65 and ≥65 years), sex (female and male), T stage (T 0–1 and T 2–4), and N stage (N 0 and N 1–3). The Kaplan–Meier survival curve showed a lower survival rate in the high-risk group than in the low-risk group based on the prognostic signature among age ≥65 years (*p* = 8.148e-06), female (*p* = 2.651e-02), male (*p* = 2.891e-04), T 0–1 (*p* = 4.945e-02), T 2–4 (*p* = 1.21e-0.3), and N1–3 (*p* = 1.087e-05) (Figure 5). However, the survival rates of patients aged <65 years and N 0 were similar between the two groups. These results demonstrate that prognostic signatures based on risk models could accurately predict prognosis relative to clinicopathological characteristics.

### Independent Prognostic Analysis of Autophagy-Related LncRNAs

**FIGURE 6:**
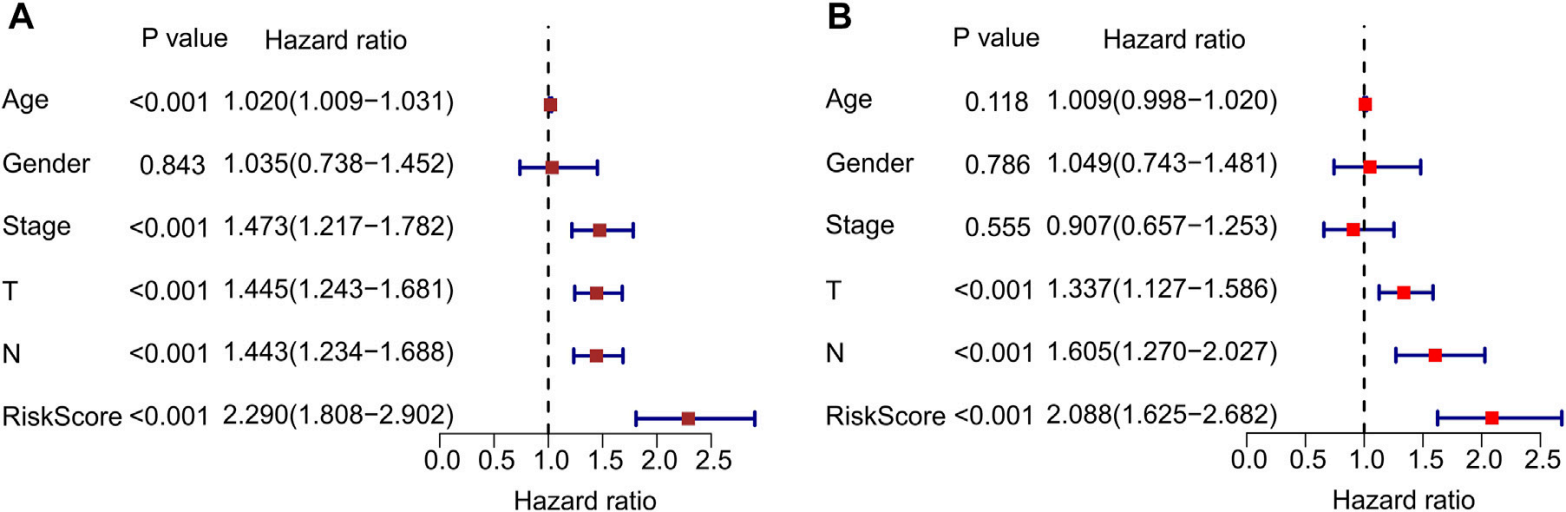
Independent prognostic analysis of autophagy-related lncRNAs. **(A)** Univariate cox regression analysis shows the correlation between overall survival and clinicopathological parameters include age, stage, T stage, N stage and the autophagy-related lncRNAs prognostic signature risk score. **(B)** Multivariate cox regression analysis reveals that T stage, N stage, and risk score are independent prognostic indicators for overall survival rates of CM patients.

Univariate and multivariate Cox regression analyses were performed to confirm that autophagy-related lncRNA prognostic signatures were independent prognostic factors in patients with CM. Univariate analysis revealed that age [hazard ratio (HR) = 1.020, *p* < 0.001], stage (HR = 1.473, *p* < 0.001), T stage (HR = 1.445, *p* < 0.001), N stage (HR = 1.443, *p* < 0.001), and risk score (HR = 2.290, *p* < 0.001) were associated with OS (Figure 6A; Supplementary Table S2). Multivariate analysis revealed that T stage [hazard ratio (HR) = 1.337, *p* < 0.001], N stage (HR = 1.605, *p* < 0.001), and risk score (HR = 2.088, *p* < 0.001) were significantly associated with OS (Figure 6B; Supplementary Table S3). Collectively, these results indicate that prognostic signatures based on autophagy-related lncRNAs are independent prognostic factors for patients with CM.

### Construction a Nomogram of Autophagy-Related LncRNAs Prognostic Features

**FIGURE 7:**
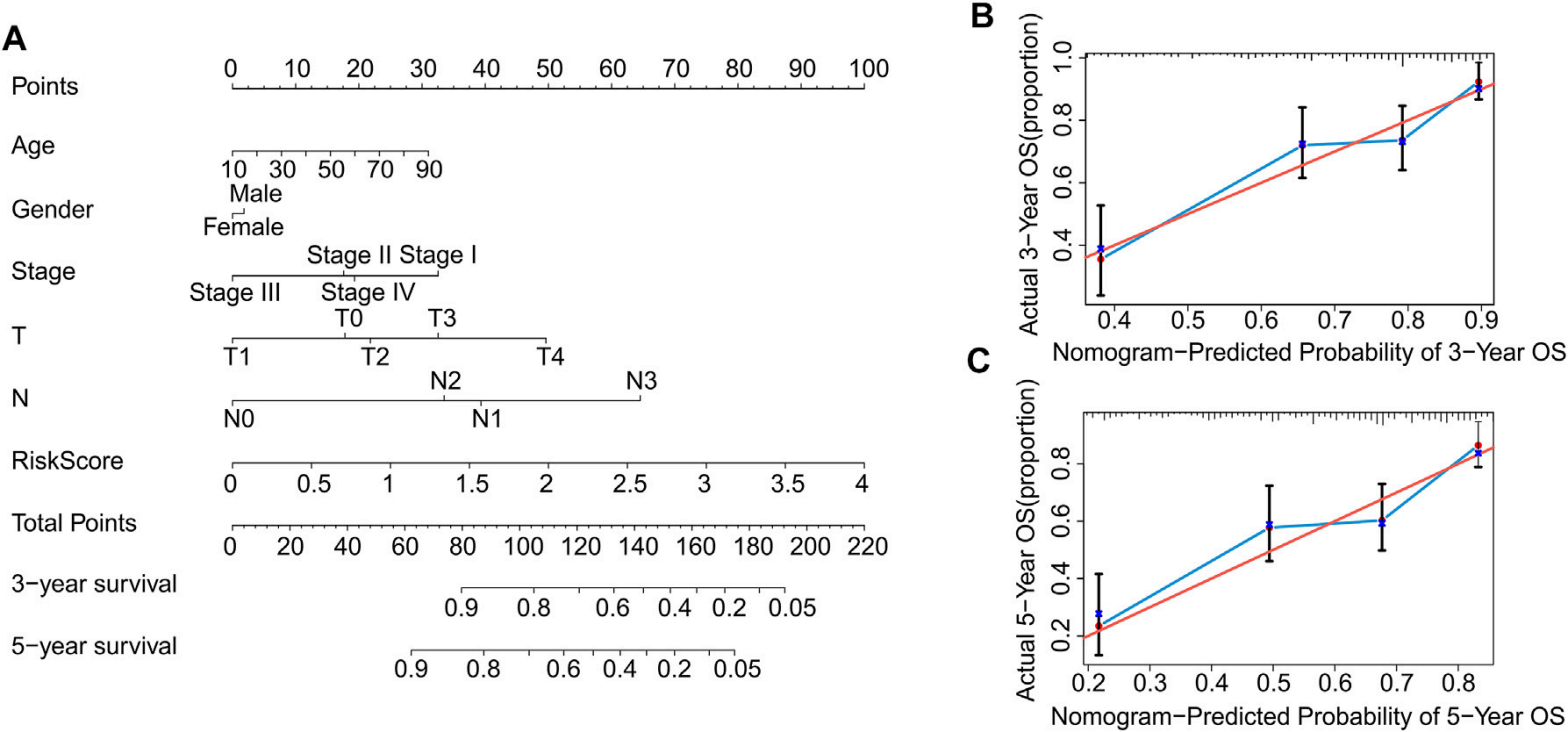
Construction and validation of prognostic nomograms based on risk scores of autophagy-related lncRNAs prognostic signatures. **(A)** Construction a nomogram using risk scores and other clinicopathological parameters (age, gender, stage, T stage, and N stage) to predict 3- and 5-years survival rate of CM patients. Calibration curve reveals the accuracy between predictive power and actual survival of **(B)** 3- and **(C)** 5-years.

Nomograms are commonly used to accurately predict the survival rate of patients with tumors. In the following study, a nomogram was constructed to accurately estimate the 3- and 5-year survival rate using the risk score and other clinicopathological features, including age, sex, stage, T stage, and N stage (Figure 7A). The consistency index (C-index) of the nomogram was 0.735. Additionally, the calibration curves indicated that the 3- and 5-year survival rates predicted using the model were consistent with the actual survival rates (Figures 7B,C). These results demonstrate that constructing a nomogram to predict the survival probability of patients using autophagy-related lncRNA prognostic signature risk scores is reliable and accurate.

### Correlation Between Risk Scores and Immune Infiltration Landscape

**FIGURE 8:**
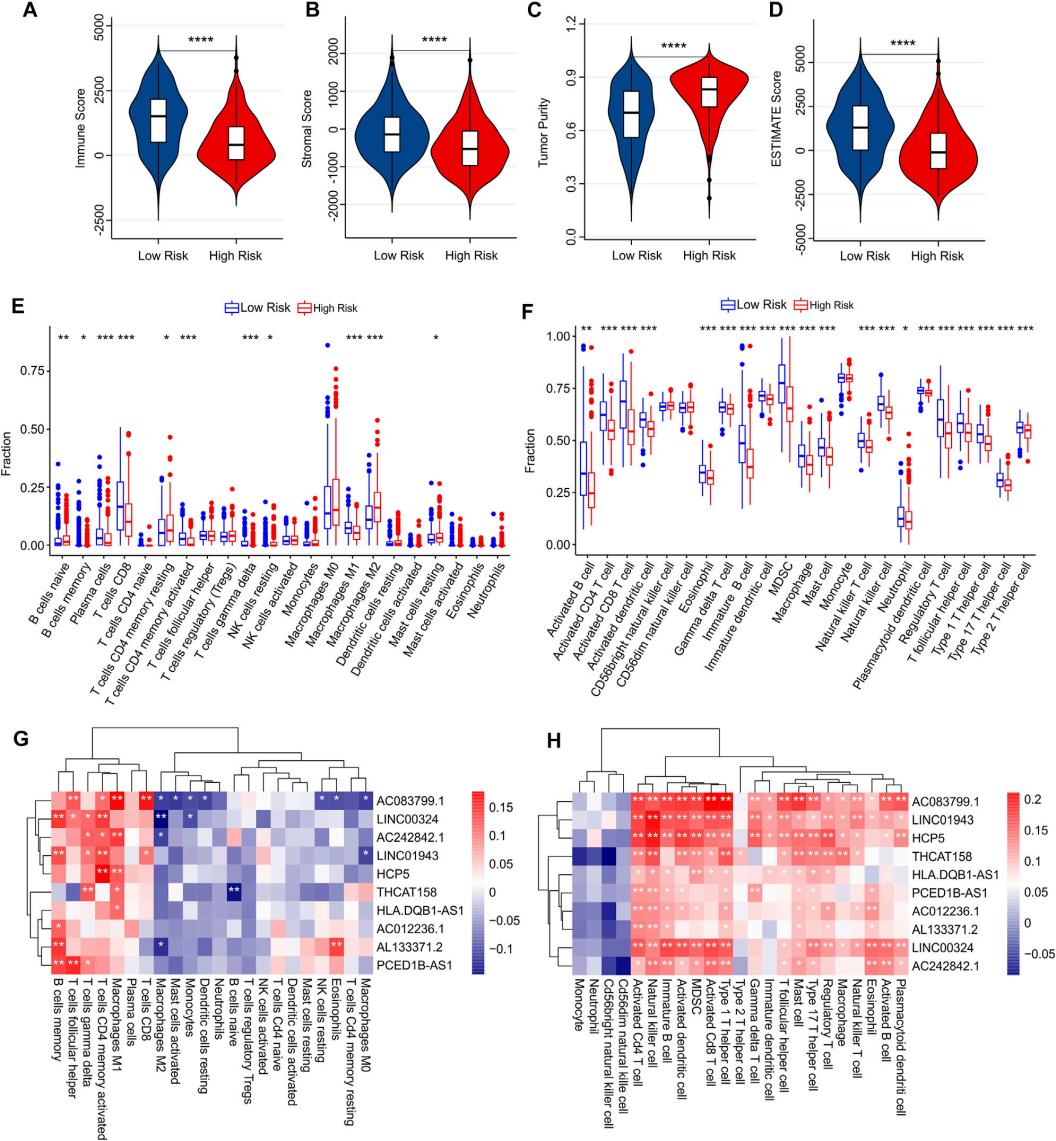
Immune infiltration analysis of CM patients in low- and high-risk groups. **(A)** Immune score. **(B)** Stromal score. **(C)** Tumor purity. **(D)** ESTIMATE score. **(E)** The fraction of 22 immune cells in low- and high-risk groups calculated by CIBERSORT algorithm. **(F)** The fraction of 23 immune cells in low- and high-risk groups calculated by ssGSEA algorithm. **(G,H)** Correlation analysis between prognostic autophagy-related lncRNAs and immune cells.

Using multiple immune assessment algorithms, we further explored the tumor microenvironment (TME) in low-risk and high-risk patients with CM. The ESTIMATE results showed that high-risk patients with CM had lower immune, stromal, and ESTIMATE scores. In particular, the tumor purity was significantly higher in the high-risk group (Figures 8A–D). Next, the CIBERSORT algorithm was used to investigate immune infiltration between low- and high-risk patients (Figure 8E). The results showed that the fractions of memory B cells; plasma cells; CD8^+^, CD4^+^ memory activated, and gamma delta T cells; and M1 macrophages were higher in the low-risk group than in the high-risk group. However, the fractions of naïve B cells, T cells CD4 memory resting, NK cells resting, M2 macrophages, resting mast cells were lower in the low-risk group than in the high-risk group. Meanwhile, the results of immune infiltration using the ssGSEA algorithm revealed that the fraction of immune cells in low-risk groups were significantly higher than high-risk groups (Figure 8F). We observed that the fractions of activated B cells, activated CD4 T cells, activated CD8 T cells, activated dendritic cells, eosinophils, gamma delta T cells, immature B cells, immature dendritic cells, MDSC, macrophages, mast cells, natural killer T cells, natural killer cells, neutrophils, plasmacytoid dendritic cells, regulatory T cells, T follicular helper cells, type 1 T helper cells, type 17 T helper cells, and type 2 T helper cells were significantly higher in the low-risk groups than in the high-risk groups.

Correlation analysis was performed to investigate the association between prognostic autophagy-related lncRNAs and the immune cells. As shown in Figure 8G, prognostic autophagy-related lncRNAs were positively correlated with memory B cells, T follicular helper cells, gamma delta T cells, activated memory CD4 T cells, M1 macrophages, and plasma cells, as determined using the CIBERSORT algorithm. Notably, prognostic autophagy-related lncRNAs were positively associated with most immune-infiltrating cells *via* the ssGSEA algorithm (Figure 8H). These results suggest that the risk model for autophagy-related lncRNAs is associated with the immune microenvironment and can indicate the immune condition of patients with CM.

### Functional Enrichment Analysis

**FIGURE 9:**
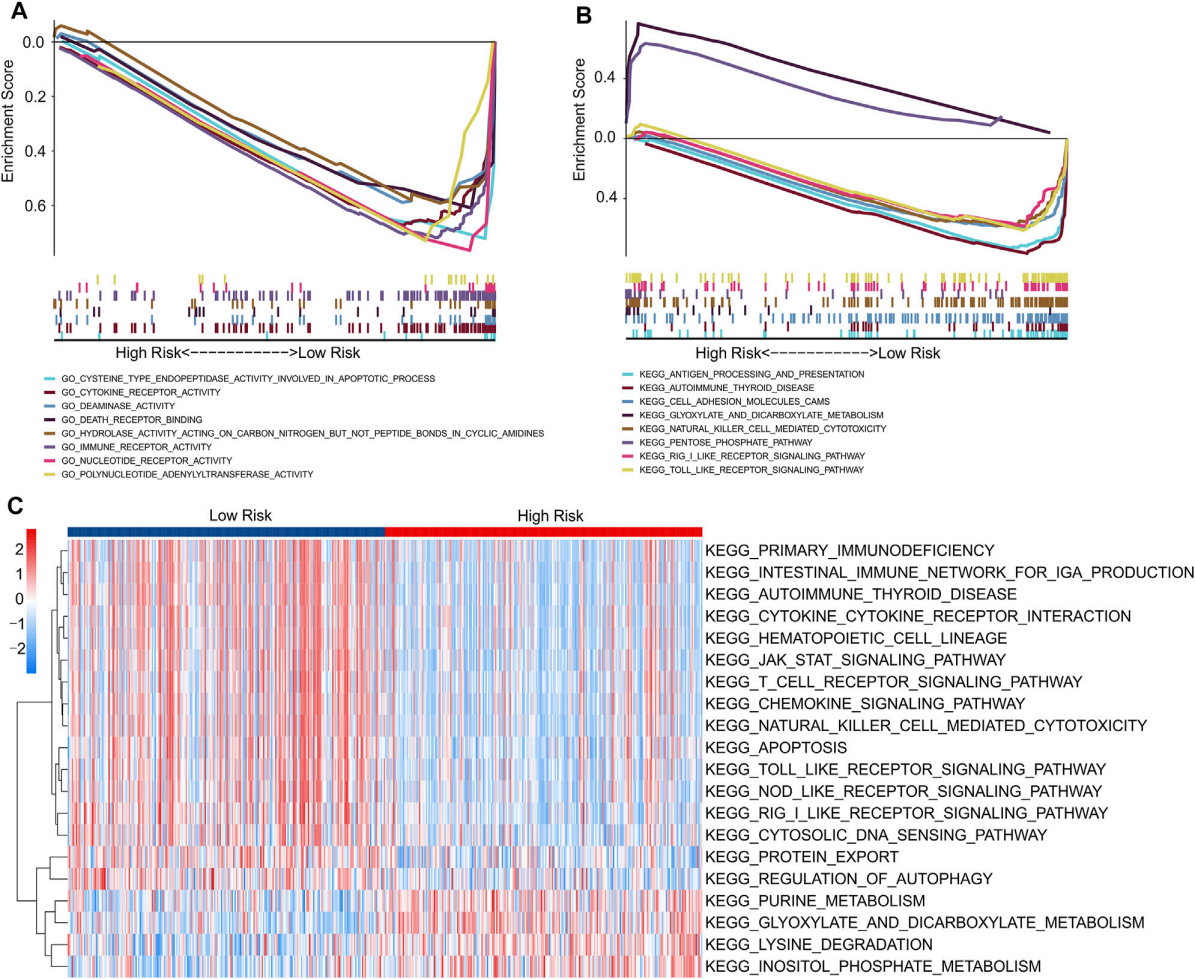
Functional enrichment analysis of low- and high-risk patients with CM. **(A)** GO analysis shows a significant enrichment of immune- and apoptotic-related process in low-risk groups. **(B)** KEGG analysis shows a significant enrichment of immune-related signaling pathways between low- and high-risk groups. **(C)** GSVA results indicate the KEGG terms of each patient in low- and high-risk groups.

Finally, functional analyses were performed to further explore the potential molecular mechanisms between the low- and high-risk groups. GSEA results of GO analysis results indicated that apoptosis-related and immune-related processes were significantly enriched in the low-risk groups, including cysteine-type endopeptidase activity involved in the apoptotic process, cytokine receptor activity, immune receptor activity, and death receptor binding (Figure 9A). In addition, the GSEA results of KEGG suggested that immune-related signaling pathways, including the Toll-like receptor signaling pathway, RIG-I-like receptor signaling pathway, and natural killer cell-mediated cytotoxicity, were enriched in the low-risk groups. Moreover, a series of metabolic pathways were significantly enriched in the high-risk groups involved in glyoxylate and dicarboxylate metabolism and the pentose phosphate pathway (Figure 9B).

We further evaluated the potential molecular mechanisms stratified by the expression level of each prognostic lncRNA. The results of KEGG analysis indicated that cancer- and autophagy-related signaling pathways were significantly enriched in the high expression of prognostic lncRNAs, including the mTOR, MAPK, p53, WNT, and ERBB signaling pathways (Supplementary Figure S2). Conversely, the GSVA results indicated that autophagy- and immune-related signaling pathways were significantly downregulated in the high-risk groups. Meanwhile, certain small-molecule metabolism signaling pathways were upregulated in the high-risk groups (Figure 9C). These results indicate that a high prognostic signature risk score correlates with autophagy and cancer, whereas a prognostic signature low-risk score correlates with enhanced immune function. These findings provide valuable insights for future investigations into potential individualized treatments for patients with CM belonging to different risk groups.

## Discussion

As one of the most common types of melanomas, CM has a poor prognosis with strong metastasis (Rebecca et al., 2020). Although immunotherapy strategies, including PD-1 inhibitors, have been gradually used to treat malignant melanoma, satisfactory clinical results have still not been achieved. Therefore, discovering new biomarkers and potential therapeutic targets is crucial for melanoma treatment (Gide et al., 2019). Constructing a risk model is a novel and practical method for predicting patient prognosis, which provides a novel perspective for clinical tumor grading. In this study, considering the significance of autophagy-related lncRNAs in CM biology, we developed a prognostic risk model based on ten autophagy-related lncRNAs and investigated the effect of lncRNAs on the immune microenvironment of patients with CM. The results suggest that the risk model constructed using the ten autophagy-related lncRNAs has a strong predictive ability to predict the prognosis of patients with CM and can indicate the immune microenvironment of patients with CM in different risk subgroups. Functional analysis results indicated that autophagy-related and immune-associated signaling pathways mediate the role of autophagy-related lncRNAs in CM. In conclusion, these results could provide a novel perspective for further treatment of patients with CM while optimizing risk stratification and helping to improve the prognosis of patients.

Recently, the construction of prognostic risk models to accurately predict patient prognosis has attracted increasing attention. Luo et al. (2020) constructed an immune prognostic model for lung adenocarcinoma, while Repo et al. (2010) assembled a novel prognostic model based on the cell cycle of patients with breast cancer, proving the significance of constructing new prognostic models for disease prediction. In this study, a novel prognostic model was constructed to accurately evaluate the survival probability of patients with CM based on ten prognostic autophagy-related lncRNAs. Notably, the constructed risk model was not influenced by other clinical characteristics, including age, sex, T stage, N stage, and stage. Independence is another critical factor for an effective prognostic risk model compared with stability. According to the univariate and multivariate regression analyses, the prognosis was associated with the risk score, indicating that the risk model was an independent indicator of CM. To further explore the reliability of the risk model, an internal analysis cohort was performed to confirm the prognosis-predicting power of the risk model. The results suggest that the risk score effectively predicts a patient’s prognosis with CM, demonstrating the considerable potential for further clinical applications, including individualized prognosis and treatment.

All lncRNAs in the risk model were associated with tumor prognosis. In the present study, ten candidate autophagy-related lncRNAs, including one risk lncRNA and nine protective lncRNAs involved in tumor progression and prognosis, were selected to construct the risk model. PCED1B-AS1 is an antisense RNAs in the PC esterase domain of the lncRNAs. PCED1B-AS1 could promote the progression of pancreatic ductal adenocarcinoma by regulating the miR-411-3p/HIF 1α axis (Yao et al., 2020). Additionally, Qin et al. (2021a) showed that PCED1B-AS1 promotes the progression of clear cell renal cell carcinoma through the miR-484/ZEB1 axis. These studies suggest that PCED1B-AS1 regulates tumorigenesis by acting as a tumor-promoting gene. However, there are no reports on the regulatory mechanism of PCED1B-AS1 in CM. In this study, we found that highly expressed PCED1B-AS1 resulted in a shorter survival probability, which is consistent with previously reported results. In addition, AC012236.1, an immune-related lncRNA, could predict the prognosis of renal clear cell carcinoma (ccRCC) (Zhang et al., 2021). HCP5 is a vital lncRNA between the *MICA* and *MICB* genes in the MHC I region and is involved in many autoimmune diseases, including malignant tumors (Qin et al., 2021b). The abnormal expression of HCP5 is closely associated with the progression and drug resistance of various tumors (Zou and Chen, 2021). In addition, LINC00324, LINC01943, and HLA-DQB1-AS1 have also been reported to be involved in the development of tumors such as hepatocellular carcinoma, osteosarcoma, and breast cancer (Wu et al., 2020; Vishnubalaji and Alajez, 2021; Wang et al., 2021). However, THCAT158, AC242842.1, AC083799.1, and AL133371.2 were not frequently reported to be associated with tumors and warrant further investigation. These findings indicate that candidate autophagy-related lncRNAs are involved in the progression of multiple types of tumors. Therefore, it is reasonable to construct a risk model to predict prognosis and stratify the risk of CM.

The tumor microenvironment (TME) plays a crucial role in tumor progression (Hinshaw and Shevde, 2019). It consists of a heterogeneous population of cancer cells, infiltrating immune cells, and stromal cells (Lei et al., 2020). Tumor-antagonizing immune cells mainly consist of effector T cells, natural killer (NK) cells, dendritic cells (DCs), and M1-polarized macrophages. Tumor-promoting immune cells mainly include regulatory T cells (Tregs) and myeloid-derived suppressor cells (MDSCs) (van den Bulk et al., 2018). With the broadening of our understanding of the molecular mechanism between autophagy and the immune system, the understanding of their relationship has gradually increased. With the assistance of adaptor proteins, autophagy can induce a series of cellular immune responses *via* pattern recognition receptors (Heath et al., 2016). Moreover, autophagy modulates innate immune signaling by eliminating endogenous inflammasomes by interfering with immune mediators such as cytokines (Puleston and Simon, 2014). These studies suggest that autophagy may mediate the immune system during tumor progression.

In the present study, the immune infiltration landscape of the low-and high-risk groups was investigated using the CIBERSORT and ssGSEA algorithms. Interestingly, the immune, ESTIMATE, and stromal scores were low in CM patients with high-risk scores. Notably, we found that the tumor purity of CM patients with high-risk scores was higher than that of patients with low-risk scores, suggesting a higher proportion of tumor cells in the tumor tissue. Generally, a high tumor purity implies a shorter survival probability and is more likely to be diagnosed as malignant (Lou et al., 2021). In this study, we found that better overall survival was correlated with lower tumor purity and higher immune score, indicating that patients with high-risk scores have worse survival status. Thus, we can reasonably infer that autophagy-related lncRNAs are associated with the immune microenvironment of patients with CM.

Exploring the signaling pathways of different risk stratification groups may contribute to understanding the regulatory mechanisms of CM. GO and KEGG analyses suggested that the differentially expressed genes between the low- and high-risk groups were mainly enriched in autophagy-related and immune-related signaling pathways. More specifically, GSVA revealed that patients with low-risk scores had a higher immune status. Moreover, the metabolic pathway, which includes purine metabolism, lysine degradation, glyoxylate and dicarboxylate metabolism, and inositol phosphate metabolism, was enriched in the high-risk group. Altogether, our findings suggest that autophagy-related lncRNAs can regulate the progression of CM by regulating immune-related signaling pathways, autophagy-related signaling pathways, and metabolism, providing a new perspective for the future treatment of CM.

However, this study had several limitations. In this study, a risk model was constructed to evaluate the prognostic value of autophagy-related lncRNAs in patients with CM using a single cohort from the public TCGA database. We further investigated the association between the risk model and immune infiltration in patients with CM. Genotype greatly impacts the immune microenvironment of patients with CM, such as *BRAF*, *NRAS*, and *NF1* mutations. Unfortunately, subgroup analyses of genotypes could not be conducted owing to sampling limitations. In addition, the immune microenvironment of CM differs at different sites. This required us to perform a large-scale sample analysis and incorporate additional risk factors to construct the risk model.

In conclusion, our findings demonstrate that the risk model based on autophagy-related lncRNAs is an independent prognostic factor distinct from other clinicopathological characteristics and can indicate the immune microenvironment of CM. Moreover, our findings provide novel insights that can be used as a reference for future targeted therapies in patients with CM.

## Data Availability

The original contributions presented in the study are included in the article/Supplementary Material, further inquiries can be directed to the corresponding author.
