# Supplementary figures and images for: A Novel Risk Model Based on Autophagy-Related LncRNAs Predicts Prognosis and Indicates Immune Infiltration Landscape of Patients With Cutaneous Melanoma
Source: Front Genet. 2022 Apr 29;13:885391. doi: 10.3389/fgene.2022.885391 (PMC9101482; doi:10.3389/fgene.2022.885391)

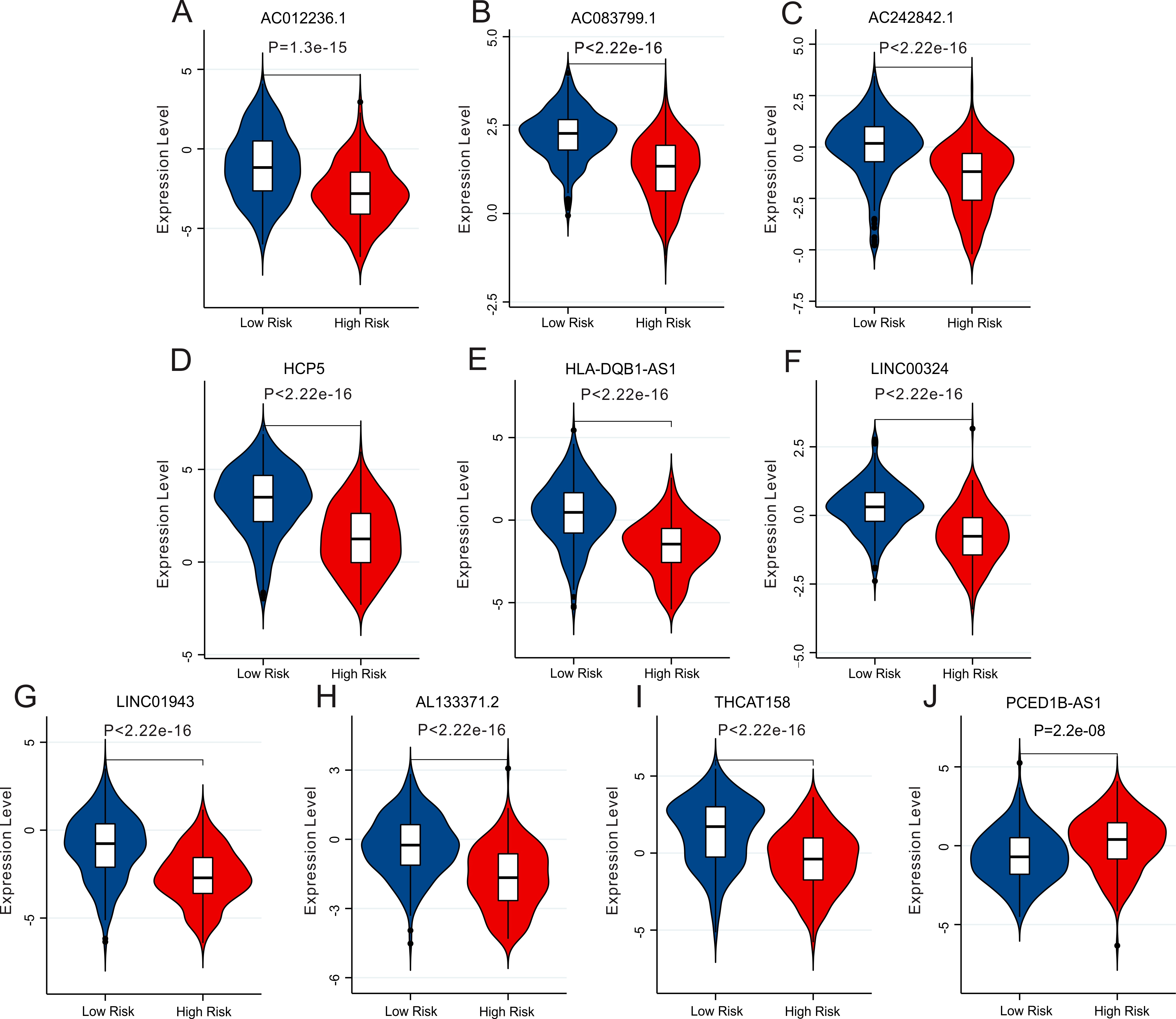

Supplement: Supplementary file 1 [file Image1.JPEG]

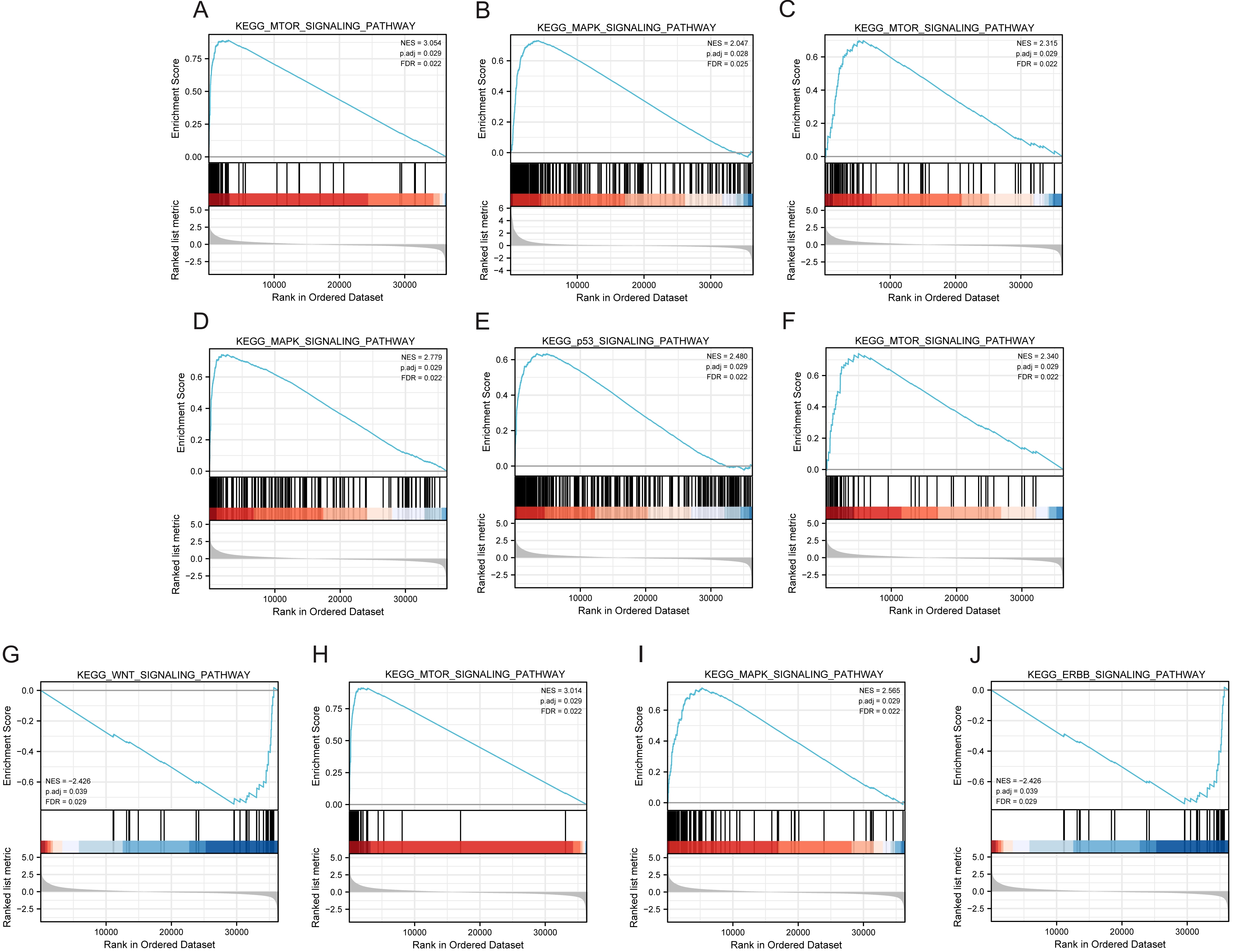

Supplement: Supplementary file 2 [file Image2.JPEG]
