# Supplementary material for: A Novel Risk Model Based on Autophagy-Related LncRNAs Predicts Prognosis and Indicates Immune Infiltration Landscape of Patients With Cutaneous Melanoma
Source: Front Genet. 2022 Apr 29;13:885391. doi: 10.3389/fgene.2022.885391 (PMC9101482; doi:10.3389/fgene.2022.885391)
Supplement: Supplementary file 3 [file DataSheet1.docx]

Supplementary Table 1. Correlation between the autophagy genes and lncRNAs in CM.

| Autophagy genes | lncRNAs | Correlation | *P* value | Regulation |
| --- | --- | --- | --- | --- |
| ERN1 | AC012236.1 | 0.304311 | 1.50E-11 | Positive |
| CCL2 | AC012236.1 | 0.306885 | 9.94E-12 | Positive |
| CDKN1B | AC012236.1 | 0.324149 | 5.52E-13 | Positive |
| CASP3 | AC012236.1 | 0.326203 | 3.87E-13 | Positive |
| CASP4 | AC012236.1 | 0.32876 | 2.47E-13 | Positive |
| DRAM1 | AC012236.1 | 0.344831 | 1.35E-14 | Positive |
| IFNG | AC012236.1 | 0.356571 | 1.44E-15 | Positive |
| IL24 | AC012236.1 | 0.378405 | 1.75E-17 | Positive |
| RGS19 | AC012236.1 | 0.387767 | 2.39E-18 | Positive |
| CASP8 | AC012236.1 | 0.392988 | 7.63E-19 | Positive |
| TNFSF10 | AC012236.1 | 0.403543 | 7.13E-20 | Positive |
| IKBKE | AC012236.1 | 0.413782 | 6.60E-21 | Positive |
| NLRC4 | AC012236.1 | 0.414098 | 6.13E-21 | Positive |
| ATG16L2 | AC012236.1 | 0.483189 | 6.32E-29 | Positive |
| PRKCQ | AC012236.1 | 0.537508 | 1.29E-36 | Positive |
| CFLAR | AC012236.1 | 0.545137 | 8.19E-38 | Positive |
| CXCR4 | AC012236.1 | 0.573475 | 1.55E-42 | Positive |
| CCR2 | AC012236.1 | 0.661491 | 1.37E-60 | Positive |
| NFKB1 | LINC00324 | 0.302018 | 2.17E-11 | Positive |
| FAS | LINC00324 | 0.307608 | 8.84E-12 | Positive |
| CALCOCO2 | LINC00324 | 0.308694 | 7.40E-12 | Positive |
| FOXO1 | LINC00324 | 0.319217 | 1.29E-12 | Positive |
| IFNG | LINC00324 | 0.320259 | 1.08E-12 | Positive |
| CDKN1B | LINC00324 | 0.333 | 1.17E-13 | Positive |
| CCL2 | LINC00324 | 0.348535 | 6.72E-15 | Positive |
| RGS19 | LINC00324 | 0.384761 | 4.56E-18 | Positive |
| CASP4 | LINC00324 | 0.390482 | 1.32E-18 | Positive |
| IKBKE | LINC00324 | 0.399164 | 1.93E-19 | Positive |
| NLRC4 | LINC00324 | 0.408121 | 2.49E-20 | Positive |
| IL24 | LINC00324 | 0.436968 | 2.20E-23 | Positive |
| CASP8 | LINC00324 | 0.448304 | 1.15E-24 | Positive |
| TNFSF10 | LINC00324 | 0.448916 | 9.79E-25 | Positive |
| CCR2 | LINC00324 | 0.464076 | 1.57E-26 | Positive |
| ATG16L2 | LINC00324 | 0.515396 | 2.56E-33 | Positive |
| CFLAR | LINC00324 | 0.52955 | 2.11E-35 | Positive |
| CXCR4 | LINC00324 | 0.539213 | 6.99E-37 | Positive |
| PRKCQ | LINC00324 | 0.597449 | 6.64E-47 | Positive |
| CDKN1B | HCP5 | 0.304473 | 1.47E-11 | Positive |
| CASP3 | HCP5 | 0.30663 | 1.04E-11 | Positive |
| ATG16L2 | HCP5 | 0.356711 | 1.40E-15 | Positive |
| CASP4 | HCP5 | 0.372105 | 6.48E-17 | Positive |
| CXCR4 | HCP5 | 0.372722 | 5.71E-17 | Positive |
| CALCOCO2 | HCP5 | 0.373096 | 5.29E-17 | Positive |
| IKBKE | HCP5 | 0.384794 | 4.53E-18 | Positive |
| NLRC4 | HCP5 | 0.406572 | 3.56E-20 | Positive |
| CASP8 | HCP5 | 0.432448 | 6.94E-23 | Positive |
| IFNG | HCP5 | 0.467911 | 5.33E-27 | Positive |
| CCR2 | HCP5 | 0.470488 | 2.56E-27 | Positive |
| DRAM1 | HCP5 | 0.494825 | 1.85E-30 | Positive |
| PRKCQ | HCP5 | 0.528062 | 3.53E-35 | Positive |
| CFLAR | HCP5 | 0.53159 | 1.04E-35 | Positive |
| TNFSF10 | HCP5 | 0.538329 | 9.59E-37 | Positive |
| APOL1 | HCP5 | 0.539374 | 6.60E-37 | Positive |
| DNAJB9 | THCAT158 | 0.318811 | 1.38E-12 | Positive |
| FAS | LINC01943 | 0.328276 | 2.69E-13 | Positive |
| CALCOCO2 | LINC01943 | 0.354776 | 2.04E-15 | Positive |
| ATG7 | LINC01943 | 0.356035 | 1.60E-15 | Positive |
| CCL2 | LINC01943 | 0.359802 | 7.67E-16 | Positive |
| CASP8 | LINC01943 | 0.362941 | 4.12E-16 | Positive |
| CXCR4 | LINC01943 | 0.381923 | 8.35E-18 | Positive |
| CASP4 | LINC01943 | 0.383886 | 5.50E-18 | Positive |
| ATG16L2 | LINC01943 | 0.395473 | 4.40E-19 | Positive |
| RGS19 | LINC01943 | 0.403947 | 6.50E-20 | Positive |
| IKBKE | LINC01943 | 0.50564 | 6.14E-32 | Positive |
| CFLAR | LINC01943 | 0.532515 | 7.51E-36 | Positive |
| CCR2 | LINC01943 | 0.5594 | 3.92E-40 | Positive |
| NLRC4 | LINC01943 | 0.581095 | 6.95E-44 | Positive |
| DRAM1 | LINC01943 | 0.588694 | 2.88E-45 | Positive |
| TNFSF10 | LINC01943 | 0.62461 | 2.55E-52 | Positive |
| PRKCQ | LINC01943 | 0.650581 | 5.04E-58 | Positive |
| APOL1 | LINC01943 | 0.651707 | 2.77E-58 | Positive |
| IFNG | LINC01943 | 0.853947 | 3.81E-135 | Positive |
| APOL1 | AC242842.1 | 0.301008 | 2.55E-11 | Positive |
| IFNG | AC242842.1 | 0.3126 | 3.90E-12 | Positive |
| NLRC4 | AC242842.1 | 0.316524 | 2.03E-12 | Positive |
| CCR2 | AC242842.1 | 0.342078 | 2.24E-14 | Positive |
| TNFSF10 | AC242842.1 | 0.348886 | 6.29E-15 | Positive |
| CASP8 | AC242842.1 | 0.355657 | 1.72E-15 | Positive |
| IL24 | AC242842.1 | 0.421882 | 9.47E-22 | Positive |
| IKBKE | AC242842.1 | 0.436296 | 2.62E-23 | Positive |
| CFLAR | AC242842.1 | 0.458411 | 7.53E-26 | Positive |
| RGS19 | AC242842.1 | 0.471146 | 2.12E-27 | Positive |
| ATG16L2 | AC242842.1 | 0.522096 | 2.72E-34 | Positive |
| PRKCQ | AC242842.1 | 0.559947 | 3.18E-40 | Positive |
| CXCR4 | AC242842.1 | 0.623913 | 3.56E-52 | Positive |
| ATG9A | AC083799.1 | -0.3828 | 6.94E-18 | Negative |
| RPTOR | AC083799.1 | -0.34392 | 1.60E-14 | Negative |
| MLST8 | AC083799.1 | -0.34392 | 1.60E-14 | Negative |
| CAPNS1 | AC083799.1 | -0.31519 | 2.53E-12 | Negative |
| GAPDH | AC083799.1 | -0.30811 | 8.14E-12 | Negative |
| CAPN1 | AC083799.1 | -0.30306 | 1.84E-11 | Negative |
| EEF2 | AC083799.1 | -0.30096 | 2.57E-11 | Negative |
| GOPC | AC083799.1 | 0.301087 | 2.52E-11 | Positive |
| CD46 | AC083799.1 | 0.301445 | 2.38E-11 | Positive |
| APOL1 | AC083799.1 | 0.306182 | 1.11E-11 | Positive |
| ATG12 | AC083799.1 | 0.311319 | 4.81E-12 | Positive |
| CDKN1B | AC083799.1 | 0.31534 | 2.47E-12 | Positive |
| CHMP2B | AC083799.1 | 0.319659 | 1.19E-12 | Positive |
| FAS | AC083799.1 | 0.319824 | 1.16E-12 | Positive |
| KLHL24 | AC083799.1 | 0.331908 | 1.42E-13 | Positive |
| SAR1A | AC083799.1 | 0.336339 | 6.40E-14 | Positive |
| IFNG | AC083799.1 | 0.358611 | 9.69E-16 | Positive |
| ATG16L2 | AC083799.1 | 0.369587 | 1.08E-16 | Positive |
| CALCOCO2 | AC083799.1 | 0.373567 | 4.80E-17 | Positive |
| ATG4C | AC083799.1 | 0.373908 | 4.47E-17 | Positive |
| CXCR4 | AC083799.1 | 0.378633 | 1.67E-17 | Positive |
| ATG4A | AC083799.1 | 0.38623 | 3.33E-18 | Positive |
| ATG5 | AC083799.1 | 0.386595 | 3.07E-18 | Positive |
| TBK1 | AC083799.1 | 0.386686 | 3.01E-18 | Positive |
| PRKCQ | AC083799.1 | 0.397122 | 3.05E-19 | Positive |
| CCR2 | AC083799.1 | 0.400287 | 1.50E-19 | Positive |
| CASP8 | AC083799.1 | 0.402276 | 9.52E-20 | Positive |
| DRAM1 | AC083799.1 | 0.420002 | 1.49E-21 | Positive |
| CASP3 | AC083799.1 | 0.422593 | 7.96E-22 | Positive |
| TNFSF10 | AC083799.1 | 0.425552 | 3.86E-22 | Positive |
| NLRC4 | AC083799.1 | 0.432131 | 7.51E-23 | Positive |
| CFLAR | AC083799.1 | 0.466825 | 7.25E-27 | Positive |
| ATG3 | AC083799.1 | 0.523394 | 1.75E-34 | Positive |
| APOL1 | HLA-DQB1-AS1 | 0.322024 | 7.96E-13 | Positive |
| IFNG | HLA-DQB1-AS1 | 0.325308 | 4.52E-13 | Positive |
| CCR2 | HLA-DQB1-AS1 | 0.32824 | 2.71E-13 | Positive |
| DRAM1 | HLA-DQB1-AS1 | 0.34061 | 2.94E-14 | Positive |
| NLRC4 | HLA-DQB1-AS1 | 0.354898 | 1.99E-15 | Positive |
| CFLAR | HLA-DQB1-AS1 | 0.361521 | 5.47E-16 | Positive |
| ATG16L2 | HLA-DQB1-AS1 | 0.36663 | 1.97E-16 | Positive |
| TNFSF10 | HLA-DQB1-AS1 | 0.371405 | 7.48E-17 | Positive |
| PRKCQ | HLA-DQB1-AS1 | 0.398979 | 2.01E-19 | Positive |
| CXCR4 | HLA-DQB1-AS1 | 0.400743 | 1.35E-19 | Positive |
| MLST8 | AL133371.2 | -0.32802 | 2.82E-13 | Negative |
| ATG7 | AL133371.2 | 0.303746 | 1.65E-11 | Positive |
| ATG5 | AL133371.2 | 0.316677 | 1.97E-12 | Positive |
| IFNG | AL133371.2 | 0.31758 | 1.70E-12 | Positive |
| CALCOCO2 | AL133371.2 | 0.331801 | 1.44E-13 | Positive |
| ERN1 | AL133371.2 | 0.334489 | 8.93E-14 | Positive |
| CASP3 | AL133371.2 | 0.370135 | 9.70E-17 | Positive |
| CDKN1B | AL133371.2 | 0.378687 | 1.65E-17 | Positive |
| NFKB1 | AL133371.2 | 0.381215 | 9.71E-18 | Positive |
| FOXO1 | AL133371.2 | 0.383945 | 5.43E-18 | Positive |
| DRAM1 | AL133371.2 | 0.385903 | 3.57E-18 | Positive |
| CASP4 | AL133371.2 | 0.408715 | 2.17E-20 | Positive |
| FAS | AL133371.2 | 0.416286 | 3.64E-21 | Positive |
| CCL2 | AL133371.2 | 0.421892 | 9.44E-22 | Positive |
| IKBKE | AL133371.2 | 0.485976 | 2.75E-29 | Positive |
| CASP8 | AL133371.2 | 0.500877 | 2.79E-31 | Positive |
| IL24 | AL133371.2 | 0.521474 | 3.35E-34 | Positive |
| RGS19 | AL133371.2 | 0.529684 | 2.02E-35 | Positive |
| TNFSF10 | AL133371.2 | 0.542289 | 2.31E-37 | Positive |
| CCR2 | AL133371.2 | 0.578307 | 2.19E-43 | Positive |
| ATG16L2 | AL133371.2 | 0.600019 | 2.15E-47 | Positive |
| NLRC4 | AL133371.2 | 0.609348 | 3.27E-49 | Positive |
| CFLAR | AL133371.2 | 0.662573 | 7.50E-61 | Positive |
| PRKCQ | AL133371.2 | 0.720596 | 1.31E-76 | Positive |
| CXCR4 | AL133371.2 | 0.740847 | 4.29E-83 | Positive |
| NFKB1 | PCED1B-AS1 | 0.320657 | 1.01E-12 | Positive |
| MTMR14 | PCED1B-AS1 | 0.327181 | 3.26E-13 | Positive |
| FAS | PCED1B-AS1 | 0.359848 | 7.60E-16 | Positive |
| CDKN1B | PCED1B-AS1 | 0.387328 | 2.62E-18 | Positive |
| APOL1 | PCED1B-AS1 | 0.39781 | 2.61E-19 | Positive |
| CASP4 | PCED1B-AS1 | 0.401602 | 1.11E-19 | Positive |
| DRAM1 | PCED1B-AS1 | 0.424345 | 5.19E-22 | Positive |
| CCL2 | PCED1B-AS1 | 0.492086 | 4.30E-30 | Positive |
| CASP8 | PCED1B-AS1 | 0.49219 | 4.17E-30 | Positive |
| IFNG | PCED1B-AS1 | 0.525801 | 7.69E-35 | Positive |
| NLRC4 | PCED1B-AS1 | 0.581296 | 6.39E-44 | Positive |
| IKBKE | PCED1B-AS1 | 0.602019 | 8.86E-48 | Positive |
| TNFSF10 | PCED1B-AS1 | 0.609702 | 2.78E-49 | Positive |
| IL24 | PCED1B-AS1 | 0.63647 | 7.40E-55 | Positive |
| CCR2 | PCED1B-AS1 | 0.661687 | 1.23E-60 | Positive |
| RGS19 | PCED1B-AS1 | 0.666161 | 1.01E-61 | Positive |
| CFLAR | PCED1B-AS1 | 0.69173 | 2.65E-68 | Positive |
| ATG16L2 | PCED1B-AS1 | 0.706586 | 1.88E-72 | Positive |
| CXCR4 | PCED1B-AS1 | 0.835368 | 5.75E-124 | Positive |
| PRKCQ | PCED1B-AS1 | 0.884991 | 8.33E-158 | Positive |

Supplementary Table 2. Univariate cox regression of clinical characteristics and risk scores.

| Variate | HR | HR.95L | HR.95H | *P* Value |
| --- | --- | --- | --- | --- |
| Age | 1.019805 | 1.008881 | 1.030847 | 0.000358 |
| Gender | 1.03489 | 0.73775 | 1.45171 | 0.842569 |
| Stage | 1.472623 | 1.217223 | 1.781613 | 6.81E-05 |
| T | 1.445359 | 1.242528 | 1.681301 | 1.80E-06 |
| N | 1.443064 | 1.233544 | 1.68817 | 4.60E-06 |
| Risk score | 2.290255 | 1.80763 | 2.901738 | 6.73E-12 |

Abbreviation HR: Hazard Ratio

Supplementary Table 3. Multivariate cox regression of clinical characteristics and risk scores.

| Variate | HR | HR.95L | HR.95H | *P* Value |
| --- | --- | --- | --- | --- |
| Age | 1.00886 | 0.997754 | 1.020091 | 0.11833 |
| Gender | 1.048853 | 0.74291 | 1.480788 | 0.786341 |
| Stage | 0.907413 | 0.657404 | 1.2525 | 0.554632 |
| T | 1.337158 | 1.127546 | 1.585863 | 0.000843 |
| N | 1.60487 | 1.270456 | 2.027309 | 7.25E-05 |
| Risk score | 2.087715 | 1.624813 | 2.682496 | 8.66E-09 |

Abbreviation HR: Hazard Ratio


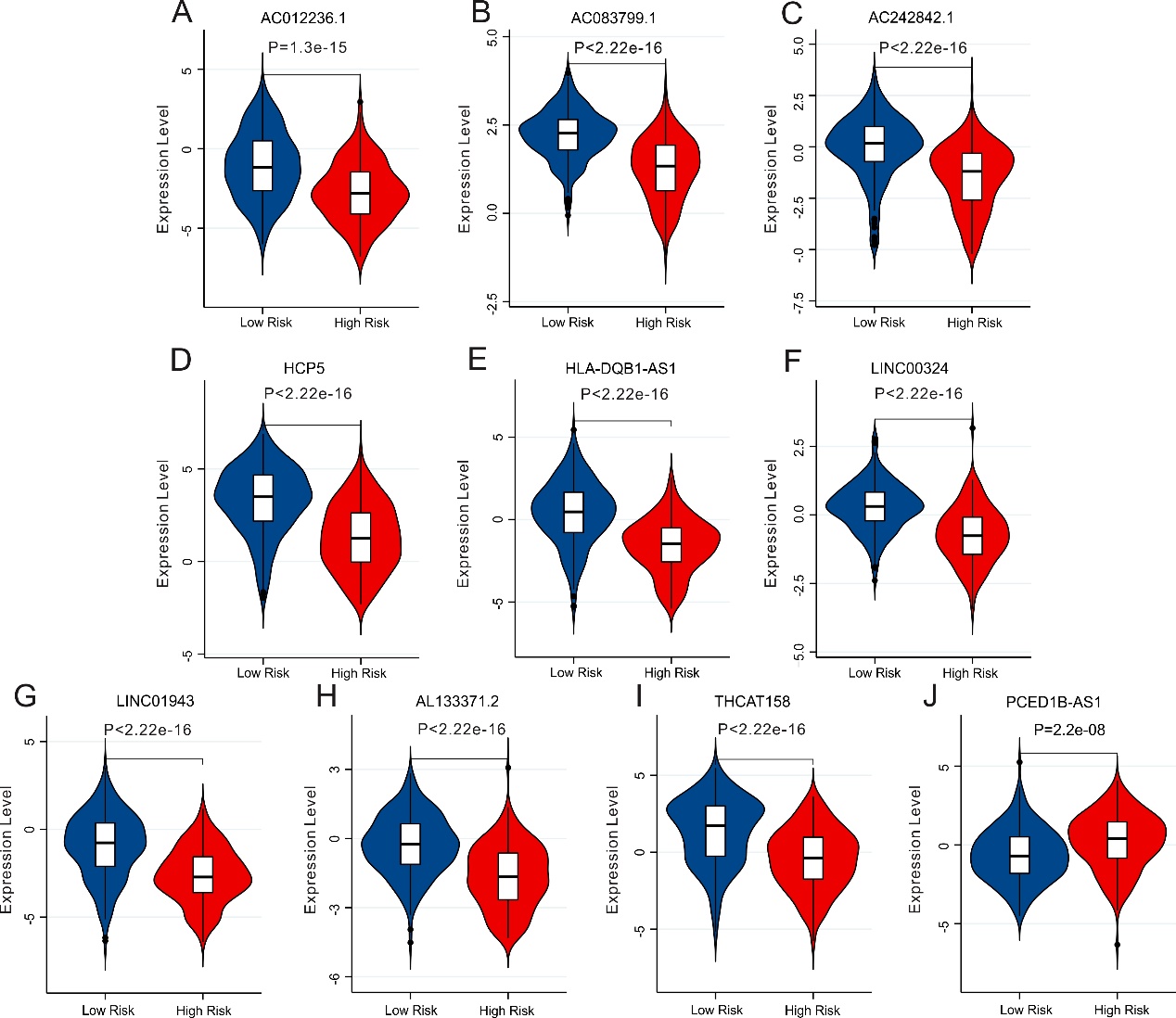


Supplementary Figure 1. The expression of ten autophagy-related lncRNAs in low- and high-risk groups. (A) AC012236.1, (B) AC083799.1, (C) AC242842.1, (D) HCP5, (E) HLA−DQB1−AS1, (F) LINC00324, (G) LINC01943, (H) AL133371.2, (I) THCAT158, (J) PCED1B−AS1. The expression of lncRNA is transformed by log_2_ (expression + 1).


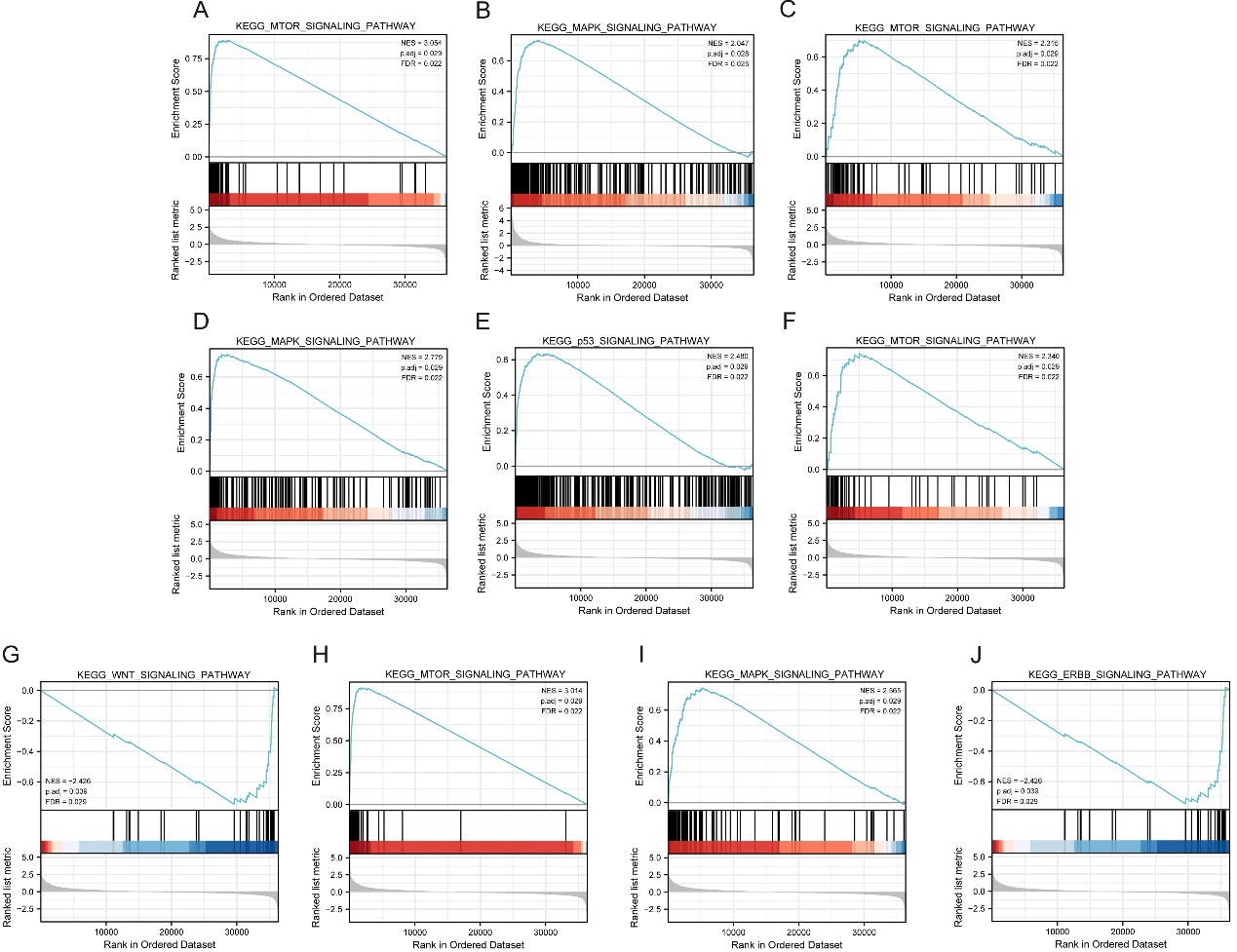
 Supplementary Figure 2. GSEA shows that the differentially expressed genes are enriched in autophagy- and cancer-related signaling pathways between low- and high-expression of each prognostic lncRNA. (A) AC012236.1, (B) LINC01943, (C) AC242842.1, (D) AC083799.1, (E) PCED1B−AS1, (F) HLA−DQB1−AS1, (G) AL133371.2, (H) THCAT158, (I) HCP5, (J) LINC00324.
